# Association between different acute stroke therapies and development of post stroke seizures

**DOI:** 10.1186/s12883-018-1064-x

**Published:** 2018-05-03

**Authors:** Jillian Naylor, Arthur Thevathasan, Leonid Churilov, Ruibing Guo, Yunyun Xiong, Miriam Koome, Ziyi Chen, Ziyuan Chen, Xinfeng Liu, Patrick Kwan, Bruce C. V. Campbell

**Affiliations:** 1 Melbourne Brain Centre, Royal Melbourne Hospital and Department of Neurology, University of Melbourne, Parkville, Melbourne, Australia; 2 The Florey Institute of Neuroscience and Mental Heath, University of Melbourne, Parkville, Melbourne, Australia; 3 Department of Neurology, Jingling Hospital, Medical School of Nanjing University, Nanjing, China; 4 Department of Neurology, The First Affiliated Hospital, Sun Yat-sen University, Guangzhou, China; 5 Department of Neurology, Royal Melbourne Hospital, Parkville, VIC 3050 Australia

**Keywords:** Ischemic stroke, Post stroke seizures, Intravenous tissue plasminogen activator, Intra-arterial thrombectomy

## Abstract

**Background:**

Epilepsy is a major complication of stroke. We aimed to establish whether there is an association between intravenous thrombolysis, intra-arterial thrombolysis and post stroke seizure (PSS) development. Improved understanding of the relationship between reperfusion therapies and seizure development may improve post-stroke monitoring and follow-up.

**Methods:**

This was a retrospective, multicentre cohort study conducted at the Royal Melbourne Hospital and Jingling Hospital Nanjing. We included patients with anterior circulation ischemic stroke admitted 2008–2015. Patients were divided into four treatment groups 1. IV-tPA only, 2. Intra-arterial therapies (IAT) only, 3. IAT + IV-tPA and 4. stroke unit care only (i.e. no IV-tPA or IAT). To assess the association between type of reperfusion treatment and seizure incidence we used multivariable logistic regression models adjusted for age, stroke severity, 3-month functional outcome and prognostic factors.

**Results:**

There were 1375 stroke unit care-only patients, of whom 28 (2%) developed PSS. There were 363 patients who received only IV-tPA, of whom 21 (5.8%) developed PSS. There were 93 patients who received IAT only, of whom 12 (12.9%) developed PSS and 112 that received both IV-tPA + IAT, of which 5 (4.5%) developed PSS. All reperfusion treatments were associated with seizure development compared to stroke unit care-only patients: IV-tPA only adjusted odds ratio (aOR) 3.7, 95%CI 1.8–7.4, *p* < 0.0001; IAT aOR 5.5, 95%CI 2.1–14.3, p < 0.0001, IAT + IV-tPA aOR 3.4, 95% CI 0.98–11.8, *p* = 0.05. These aORs did not differ significantly between treatment groups (IV-tPA + IAT versus IV-tPA *p* = 0.89, IV-tPA + IAT versus IAT, *p* = 0.44).

**Conclusions:**

Patients receiving thrombolytic or intra-arterial reperfusion therapies for acute ischemic stroke are at higher risk of epilepsy and may benefit from longer follow-up. No evidence for an additive or synergistic effect of treatment modality on seizure development was found.

**Electronic supplementary material:**

The online version of this article (10.1186/s12883-018-1064-x) contains supplementary material, which is available to authorized users.

## Background

With greater public awareness of the importance of early stroke recognition and more efficient treatment delivery (e.g. code stroke, telemedicine), there has been a welcome increase in the proportion of patients arriving at hospital within the timeframe to be eligible for reperfusion therapies [1]. Numerous randomized controlled trials have demonstrated the superiority of reperfusion therapies, including endovascular thrombectomy and IV-tPA, over standard treatment for acute ischemic stroke patients with large artery occlusion [2–6]. These advances have contributed to a 68% increase in the number of stroke survivors between 1990 and 2010 [7]. Given the improved immediate outlook of acute stroke, there is a need to understand whether these interventions also affect long term complications.

Epilepsy is one of the major complications of stroke. Post-stroke epilepsy poses a considerable burden to stroke survivors and, even when well-controlled with medications, negatively impacts their quality of life [8]. Seizures develop in 2–14% of patients who have had an ischemic stroke [9]. Such wide variation in the reported incidence and prognostic factors [10] has been attributed to differences in follow-up duration, definition and classification of seizures, and characteristics of the study population [11].

Whether the recent advances in reperfusion therapies for acute ischemic stroke have influenced the incidence of seizure development has not been well studied. Two studies have found that thrombolysis increases the likelihood of acute symptomatic seizures, within 7 days, post ischemic stroke, [12, 13] whilst others have shown no association [14]. Few have examined post-stroke seizures following intra-arterial therapies (IAT).

This study aimed to investigate whether there is an association between different acute stroke treatments and post stroke seizure development. A better understanding of the relationship between acute stroke therapies and the development of seizures may lead to improved post stroke monitoring and follow-up.

## Methods

### Setting

This was a retrospective, multicentre cohort study conducted at the Royal Melbourne Hospital and Jingling Hospital, Nanjing. Subjects at both centres were identified from prospectively maintained clinical databases of patients admitted with an ischemic stroke. The Royal Melbourne Hospital, located in Victoria, Australia, provides IV-tPA therapy to acute ischemic stroke patients who arrive to the hospital within 4.5 h of stroke onset. It also serves as the state-wide referral centre for intra-arterial therapies, including endovascular thrombectomy and intra-arterial urokinase. Jingling Hospital is located in Jiangsu province, China, where most patients received stroke unit care only. Ethical approval of the study was granted by the Melbourne Health Human Research Ethics Committee (project number QA2010089) and patient consent was waived.

### Patient groups

We included patients with anterior circulation ischemic stroke admitted to the two hospitals between 2008 and 2015. Patients with a history of epilepsy or seizures prior to their stroke were excluded. Patients included were divided into 4 groups based on the type of acute reperfusion treatment received: 1. IV-tPA only, 2. IAT only, 3. IAT + IV-tPA and 4. stroke unit care only (i.e. no IV-tPA or IAT). For the purpose of analyses, patients receiving ‘stroke unit care only’ were regarded as controls.

### Clinical data collection

Clinical data was collected and entered into the databases at both centres when the patients were admitted to the emergency department, transferred to stroke wards and returned to stroke follow-up clinics. Data included patient demographics, age, sex, pre-morbid modified Rankin score (mRS), admission National Institutes of Health Stroke Scale (NIHSS) score, admission blood pressure and stroke risk factors such as hypertension, atrial fibrillation, diabetes, dyslipidemia, previous stroke or transient ischemic attack (TIA) and smoking. Clinical follow-up information included the modified Rankin Scale (mRS) at 3-months post onset with good outcome defined as mRS 0–2. Hemorrhagic transformation was assessed using the ECASS classification [15] on follow-up 24 h CT brain imaging, blinded to seizure data collection.

### Seizure follow-up

Patients with post stroke seizures were identified if they experienced seizures up to 2 years from stroke onset. This cut-off was chosen based on previous findings that suggested that the highest risk of seizure development was within the first year [16]. Occurrence of post stroke seizures was ascertained by reviewing follow-up medical records and via telephone interview using a questionnaire from previous studies [14, 17–19], modified from a validated screening questionnaire [20]. This questionnaire was translated into Chinese for use at the Jingling Hospital Nanjing. Events were recorded as seizures if the symptoms included motor or autonomic components, with or without impairment of consciousness, as defined by the International League Against Epilepsy [21].

### Statistical analyses

To assess homogeneity between the two sites, the seizure occurrence within 2 years post-stroke between the control groups across the two sites was compared using Fisher’s Exact test with corresponding effect estimated as Odds Ratios (OR) with 95% confidence interval (95%CI). When no significant difference in seizure incidence between the two sites was found, the control groups were combined.

The demographic, clinical and risk factor characteristics for the control group, IAT only, IV-tPA only and IAT + IV-tPA combined groups were summarised as median (IQR) for continuous characteristics and as counts (proportions) for categorical characteristics, and compared using either Kruskall-Wallis test or Fisher’s Exact test depending on the nature of the distribution.

To assess the association between the types of treatment (reperfusion: IAT only, IV-tPA only, IAT + IV-tPA; with controls) and seizure occurrence within 2 years, logistic regression modelling with seizure occurrence as an output and treatment groups as inputs was used. The analysis was adjusted for the following a priori chosen covariates known to be associated with post stroke seizures: age, baseline NIHSS and 3 month mRS [16, 22–26]. Corresponding effect sizes were summarised as adjusted ORs with 95%CI. For robustness analysis, extra covariates that demonstrated statistically significant association with seizure occurrence on univariate analyses were subsequently included in the model.

To further investigate the robustness of the modelling outcomes, we performed a sensitivity analysis to assess the potential association between thrombectomy and seizures with a control group more closely matching the patients who receive thrombectomy. We used a selection criterion of NIHSS≥6 as per AHA/ASA guidelines for thrombectomy eligibility [27]. We additionally performed a sensitivity analysis including only patients with an NIHSS> 8 to further increase specificity for large vessel occlusion [28].

Statistical analyses were performed using STATA IC (v13.1, StataCorp, College Station, TX, USA), *p*-value < 0.05 was treated as indicative of statistical significance.

## Results

### Patient characteristics

A total of 1943 patients with anterior circulation ischemic stroke were included in the analysis (757 from Melbourne and 1186 from Nanjing). The overall incidence of post-stroke seizures within 2 years was 3.3% (65/1943). No significant difference in seizure occurrence was identified between the Melbourne (1/189, 0.53%) and the Nanjing (27/1186, 2.3%) stroke unit care-only patients (OR 0.24, 95%CI 0.0–1.4, *p* = 0.1). Consequently, these patients were combined into a single control group for the multivariable regression models to assess the influence of treatment on seizure development. In the combined cohort, 1375 patients were controls, of which 27 (2, 95%CI 1.3–2.8%) developed post stroke seizures. There were 363 patients treated with IV-tPA only. Of these, 21/363 (5.8, 95%CI 3.6–8.7%) patients developed post stroke seizures. There were 93 patients who received IAT only, with 12 (12.9, 95%CI 6.8–21.4%) developing post stroke seizures. There were 112 patients treated with IAT and IV-tPA, of whom 5 (4.5, 95%CI 1.5–10%) developed post stroke seizures.

There was a significant difference in baseline NIHSS across treatment groups median (IQR): IAT + IV-tPA = 17 (13–21), IAT = 18 (13–21), IV-tPA only = 10 (6–17), control = 2 (0–7), *p* = 0.0001. There was a significant difference in age at stroke across the treatment groups median (IQR): IAT + IV-tPA = 70 (59–78), IAT = 67 (54–77), IV-tPA only = 74 (65–77) control = 62 (52–70), *p* = 0.0001. There was a significant difference in mRS 0–2 at 3 months across the treatment groups: IAT + IV-tPA = 59/112 (52.7%), IAT = 36/93 (38.7%), IV-tPA only = 191/363 (52.6%) control = 839/1375 (61.0%), *p* < 0.0001. Baseline characteristics are detailed in Table 1.

**Table 1:** Baseline demographics and stroke risk factors across treatment group

	IAT + IV-tPA, *n* = 112	IAT only *n* = 93	IV-tPA only, *n* = 363	Control, *n* = 1375	*p*-value
Age (median, IQR)	70 (59–78)	67 (54–77)	74 (65–82)	62 (52–70)	0.0001^a^
Female Sex (n,%)	45 (40.2)	50 (54)	175 (48.2)	477 (34.7)	< 0.001^b^
Systolic Blood Pressure (median, IQR)	- ^x^	- ^x^	150 (133–167)	136 (128–150)	0.0001^a^
Diastolic Blood Pressure (median, IQR)	- ^x^	- ^x^	80 (70–90)	80 (72–88)	0.50^a^
NIHSS baseline (median, IQR)	17 (13–21)	18 (13–21)	10 (6–17)	2 (0–7)	0.0001^a^
Hypertension (n,%)	66 (58.9)	42 (45)	264 (73.0)	823 (59.8)	< 0.001^b^
Diabetes (n,%)	19 (17.0)	19 (20.4)	106 (29.2)	299 (21.7)	0.05^b^
Dyslipidaemia (n,%)	33 (29.5)	28 (30)	191 (52.6)	53 (4)	< 0.001^b^
Smoking (n,%)	- ^x^	- ^x^	88 (24)	438 (32)	0.001^b^
Atrial Fibrillation (n,%)	43 (38.4)	31 (33.3)	115 (31.7)	114 (8.3)	< 0.001^b^
Hemorrhagic Transformation (n,%)	28 (25)	28 (30)	63 (17)	-^x^	0.001^b^
3-month mRS (0–2) (n,%)	59 (53)	36 (39)	191 (53)	839 (61)	< 0.001^b^
Post Stroke Seizures (n,%)	5 (4.5)	12 (12.9)	21 (5.8)	28 (2)	< 0.001^b^

^a^Kruskall-Wallis test

^b^Fisher’s Exact test

^x^unavailable

### The association of IAT, IV-tPA and IAT + IV-tPA with post stroke seizure development

**Fig. 1:**
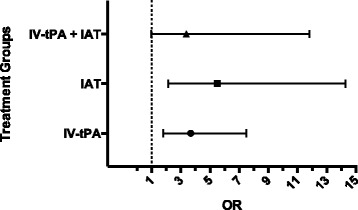
Forest plot of treatment effects on seizure development post stroke. The effects shown are ORs with 95% CIs (adjusted for age, baseline NIHSS and 3 month mRS) using stroke unit care only group as a reference. No significant difference in seizure development post stroke between IAT, IV-tPA and IV-tPA + IAT treatment groups (IV-tPA + IAT versus IV-tPA *p* = 0.89, IV-tPA + IAT versus IAT, *p* = 0.44)

The univariate analysis for baseline variables age, NIHSS at baseline and the 3-month mRS of 0-2 and their association with post stroke seizures are provided in Table 2. In multivariable logistic regression adjusted for baseline NIHSS, age, and mRS at 3 months, administration of IV-tPA (without IAT) was significantly associated with increased odds of seizure development: adjusted OR 3.7, 95%CI 1.8–7.4, *p* < 0.0001. Similarly, there was an independent association between treatment with IAT only and increased odds of seizure development compared to controls: adjusted OR 5.5, 95%CI 2.1–14.3, *p* < 0.0001. There was also a trend towards increased seizure risk in patients treated with combined IAT + IV-tPA compared to controls: OR 3.4, 95%CI 0.98–11.8, *p* = 0.05 (Fig. 1). For the purpose of robustness analysis, we included extra baseline adjustment covariates and risk factors that were significantly associated with seizure development in univariate analysis and were recorded for all treatments: sex, hypertension, atrial fibrillation and dyslipidemia (Table 1). Including these variables as extra covariates increased the collinearity in the regression model and rendered some standard error estimates less stable, but the adjusted estimates for the effects for individual treatment groups remain quantitatively similar: IAT only OR 6.6, 95%CI 2.5–17.8, *p* < 0.001, IV-tPA only OR 2.9, 95%CI 2.3–10.7, p < 0.001, IV-tPA+ IAT OR 4.3, 95%CI 1.2–15.3, *p* = 0.03. Consistently with largely overlapping confidence intervals, the odds of post stroke seizures in the three treatment groups did not differ (IV-tPA + IAT versus IV-tPA *p* = 0.89, IV-tPA + IAT versus IAT, *p* = 0.44, Fig. 1).

**Table 2:** Univariate Analysis for baseline variables and the association with post stroke seizures

	IAT + IV-tPA, *n* = 112 OR, 95% CI, *p*-value	IAT only *n* = 93 OR, 95% CI, *p*-value	IV-tPA only, *n* = 363 OR, 95% CI, *p*-value	Control, *n* = 1375 OR, 95% CI, *p*-value
Age	1.0, 0.94–1.1, *p* = 0.9	0.94, 0.9–0.98, *p* = 0.003	1.0, 0.96–1.0, *p* = 0.95	0.99, 0.96–1.0, *p* = 0.33
NIHSS baseline	1.1, 0.9–1.3, *p* = 0.36	1.0, 0.92–1.1, *p* = 0.98	1.1, 1.0–1.1, *p* = 0.05	1.1,1.0–1.1, *p* < 0.0001
3-month mRS (0–2)	0.13, 0.01–1.3, *p* = 0.08	0.52, 0.12–2.2, *p* = 0.37	0.37, 0.14–0.97, *p* = 0.04	0.27, 0.12–0.62, *p* = 0.002

For the sensitivity analysis including only patients with an NIHSS ≥6 (to better match the controls with patients eligible for thrombectomy [27]), the results of the logistic regression were similar to the previous results: IV-tPA verses controls OR 3.2, 95%CI 1.4–7.4, *p* = 0.005, IAT verses controls OR 4.6, 95%CI 1.6–13.0, *p* = 0.004, IV-tPA + IAT versus controls OR 3.2, 95%CI 0.84–12.1, *p* = 0.09.

For the sensitivity analysis including only patients with an NIHSS> 8 (to further increase specificity for large vessel occlusion [28]) the results of the logistic regression were similar to the previous results: IV-tPA versus controls OR 3.9, 95%CI 1.5–9.7, p = 0.004, IAT versus controls OR 5.3, 95%CI 1.9–15.3, *p* = 0.002, IV-tPA + IAT versus controls OR 4.0, 95%CI 1.03–16.2, *p* = 0.045. Additional file 1 for the univariate and multivariable logistic regression has been presented.

## Discussion

This study has demonstrated that acute stroke reperfusion therapies are significantly associated with seizure development. Specifically, we showed that in patients treated with IV-tPA only, independent of age, baseline stroke severity, stroke outcome and other baseline variables, there was a greater than threefold increase in the likelihood of developing seizures in comparison to controls. This was a similar effect to those patients treated with IV-PA+ IAT. We also found that in patients treated with IAT only, there was a greater than fivefold increase in the likelihood of developing seizures in comparison to controls. However, there was no evidence for an additive or synergistic effect of treatment modality.

There have been two studies that have reported an association between thrombolysis with IV-tPA and increased likelihood of acute symptomatic seizures, within 7 days, after an ischemic stroke [12, 13]. We have shown that the risk of seizures extends further than the acute symptomatic period. In this study, we found the development of seizure occurred over a 24-month period, and recommend longer-term follow-up of these higher risk patients. This could significantly improve the detection of late onset complications of stroke.

Our analysis was adjusted for prognostic factors for seizure development as well as factors that were significantly different between treatment groups, suggesting that the increased risk of post stroke seizure development may be inherent to the treatment itself. A number of potential mechanisms may account for this. Firstly, sudden changes in cerebral perfusion have been described to cause a clinical syndrome that includes seizures [29–31]. When perfusion is improved by revascularization procedures, it is suggested a cascade of inflammatory responses causes the reestablishment of brain circulation, contributing to the development of the reperfusion syndrome and subsequent seizures, with seizures a sign of good reperfusion [32]. Given that early onset seizures are a risk factor for later onset seizures and epilepsy development, potentially the benefit of reperfusion from acute stroke therapies is concomitantly increasing the risk of seizure development in these populations.

Although IV-tPA has been reported to have some neurotoxicity [33], the greater odds of developing seizures from any IAT treatment compared to any IV-tPA treatment would argue against a specific effect of IV-tPA and perhaps reperfusion itself is the issue. In our study, we showed that patients who underwent any intra-arterial therapy were significantly more likely to develop seizures than those with any IV-tPA. Of interest, there was a significant difference in rates of hemorrhagic transformation between the treatment groups (IAT, IV-tPA and IAT + IV-tPA). This was also suggested in a review of rates of HT in major clinical trials of ischemic stroke interventions where they found observable trends in increased HT in patients with intra-arterial therapies [34]. Potentially, the greater likelihood of developing post stroke seizures in patients undergoing IAT rather than IV-tPA may be due to increased rates of HT within this treatment group. An analysis of post stroke seizures due to HT across different treatment groups is a planned follow up analysis.

A limitation of this study is the retrospective design with the potential of bias towards identifying seizures in those patients receiving reperfusion therapies given that they may have received more monitoring in the stroke unit than those patients without reperfusion therapies. However, given that all patients were contacted via a phone call questionnaire we believe this would have minimised any bias towards treatment groups. Another limitation is that we do not understand the potential ethnicity-treatment interaction without a treated group from Nanjing. We cannot discount the potential effect of the treatment itself differing due to ethnicity, although we do not expect this. Future studies should incorporate a treatment group from separate ethnicities to examine a possible treatment-ethnicity interaction. Additionally, due to the low incidence of post stroke seizure development, future larger studies are required to improve the precision of incidence estimates. Finally, we were unable to perform a subanalysis on patients with large vessel occlusion as vessel occlusion status was not available in our control population. Even with adjustment for baseline NIHSS, initial stroke severity may still be contributing to the higher odds of seizures in the treated patients. It would be of interest to perform a future analysis specifically in large vessel occlusion patients.

## Conclusion

Patients undergoing reperfusion therapies with IV-tPA and IAT were at considerably higher risk of post-stroke epilepsy than control patients. However, no additional effect from combined treatment was evident in this dataset. This association persisted despite adjustment for differences in stroke severity and other prognostic variables. We conclude that patients undergoing IAT and/or IV-tPA in acute ischemic stroke may benefit from longer stroke follow-up for late complications of treatment such as epilepsy.

## Additional file

Additional file 1: **Table S1.** Univariate logistic regression with baseline risk factors (age, NIHSS and mRS02 at 90 days) and treatment for seizure outcome. **Table S2.** Logistic regression model with treatment groups plus age for seizure outcome. **Table S3.** Logistic regression model with treatment groups plus NIHSS for seizure outcome. **Table S4.** Logistic regression model with treatment groups plus mRS02 for seizure outcome. **Table S5.** Logistic regression model with treatment groups unadjusted. **Table S6.** Logistic regression model with treatment groups adjusted for age, NIHSS and mRS02. **Table S7.** Median (IQR) of the baseline NIHSS and number (percentage) of mRS (0–2) in the sensitivity analysis NIHSS ≥ 6. **Table S8.** Median (IQR) of the baseline NIHSS and number (percentage) of mRS (0–2) in the sensitivity analysis NIHSS > 8. (DOCX 63 kb)

## Abbreviations

95%CI: 95% confidence interval
ECASS: European cooperative acute stroke study
HT: Hemorrhagic Transformation
IAT: Intra-arterial therapy
IV-tPA: Intravenous-tissue plasminogen activator
mRS: modified Rankin scale
OR: Odds ratio
