# Supplementary material for: Association between different acute stroke therapies and development of post stroke seizures
Source: BMC Neurol. 2018 May 3;18:61. doi: 10.1186/s12883-018-1064-x (PMC5932812; doi:10.1186/s12883-018-1064-x)
Supplement: Supplementary file 1 — Table S1. Univariate logistic regression with baseline risk factors (age, NIHSS and mRS02 at 90 days) and treatment for seizure outcome. Table S2. Logistic regression model with treatment groups plus age for seizure outcome. Table S3. Logistic regression model with treatment groups plus NIHSS for seizure outcome. Table S4. Logistic regression model with treatment groups plus mRS02 for seizure outcome. Table S5. Logistic regression model with treatment groups unadjusted. Table S6. Logistic regression model with treatment groups adjusted for age, NIHSS and mRS02. Table S7. Median (IQR) of the baseline NIHSS and number (percentage) of mRS (0–2) in the sensitivity analysis NIHSS ≥ 6. Table S8. Median (IQR) of the baseline NIHSS and number (percentage) of mRS (0–2) in the sensitivity analysis NIHSS > 8. (DOCX 63 kb) [file 12883_2018_1064_MOESM1_ESM.docx]

Supplementary Material

Table 1: Univariate logistic regression with baseline risk factors (age, NIHSS and mRS02 at 90 days) and treatment for seizure outcome

|  | OR (95% CI), p-value |
| --- | --- |
| Age | 1.0 (0.97-1.0), p=0.43 |
| NIHSS | 1.09 (1.06-1.1), p<0.001 |
| mRS 0-2 at 90 days | 0.3 (0.16-0.48), p<0.001 |
| IV-tPA only | 2.1 (1.3-3.7), p=0.005 |
| IAT-only | 5.02 (2.6-9.8), p<0.0001 |
| IV-tPA + IAT | 1.37 (0.54-3.5), p=0.5 |

Table 2: Logistic regression model with treatment groups plus age for seizure outcome

|  | OR (95% CI), p-value |
| --- | --- |
| IV-tPA only | 3.8 (2.1-7.12), p<0.001 |
| IAT only | 7.9 (3.8-16.2), p<0.001 |
| IV-tPA + IAT | 2.7 (1.0-7.1), p=0.05 |
| Age | 0.98 (0.96-1.0), p=0.031 |

Table 3: Logistic regression model with treatment groups plus NIHSS for seizure outcome

|  | OR (95% CI), p-value |
| --- | --- |
| IV-tPA only | 2.0 (1.06-3.7), p=0.031 |
| IAT only | 3.3 (1.46-7.56), p=0.004 |
| IV-tPA + IAT | 1.13 (0.4-3.18), p=0.82 |
| NIHSS | 1.07 (1.0-1.1), p<0.001 |

Table 4: Logistic regression model with treatment groups plus mRS02 for seizure outcome

|  | OR (95% CI), p-value |
| --- | --- |
| IV-tPA only | 2.8 (1.56-5.1), p=0.001 |
| IAT only | 5.9 (2.7-12.8), p<0.001 |
| IV-tPA + IAT | 2.9 (0.97-8.5), p=0.057 |
| mRS 0-2 at 90 days | 0.37 (0.18-0.55), p<0.001 |

Table 5: Logistic regression model with treatment groups unadjusted

|  | OR (95% CI), p-value |
| --- | --- |
| IV-tPA only | 3.1 (1.7-5.5), p<0.001 |
| IAT only | 7.4 (3.6-15.1), p<0.001 |
| IV-tPA + IAT | 2.3 (0.88-6.2), p=0.088 |

Table 6: Logistic regression model with treatment groups adjusted for age, NIHSS and mRS02

|  | OR (95% CI), p-value |
| --- | --- |
| IV-tPA only | 3.7 (1.8-7.4), p<0.0001 |
| IAT only | 5.5, (2.1-14.3), p<0.0001 |
| IV-tPA + IAT | 3.4, (0.98-11.8), p=0.05 |
| Age | 0.97 (0.95-0.99), p=0.004 |
| NIHSS | 1.04 (1.0-1.08), p=0.032 |
| mRS 0-2 at 90 days | 0.35 (0.18-0.67), p=0.02 |

Table 7: Median (IQR) of the baseline NIHSS and number (percentage) of mRS (0-2) in the sensitivity analysis NIHSS≥6

|  | NIHSS≥6 | | | |
| --- | --- | --- | --- | --- |
|  | IV-tPA-only  n=299 | IAT-only  n= 85 | IV-tPA+IAT  n= 108 | Control  n= 397 |
| mRS 0-2 at 90 days (n,%) | 129 (43) | 34 (40) | 76 (70) | 25 (6.3) |
| NIHSS (median, IQR) | 13 (9-18) | 19 (14-22) | 17 (14-21) | 11 (8-15) |

Table 8: Median (IQR) of the baseline NIHSS and number (percentage) of mRS (0-2) in the sensitivity analysis NIHSS>8

|  | NIHSS>8 | | | |
| --- | --- | --- | --- | --- |
|  | IV-tPA-only  n=277 | IAT-only  n=74 | IV-tPA+IAT  n=74 | Control  n=285 |
| mRS 0-2 at 90 days (n,%) | 97 (35) | 30 (41) | 51 (69) | 3 (1.1) |
| NIHSS (median, IQR) | 16 (12-20) | 19 (14-22) | 18 (15-22) | 14 (11-18) |
